# Internet-Supported Physical Exercise Training for Persons with Multiple Sclerosis—A Randomised, Controlled Study

**DOI:** 10.3390/ijms17101667

**Published:** 2016-09-30

**Authors:** Alexander Tallner, René Streber, Christian Hentschke, Marc Morgott, Wolfgang Geidl, Mathias Mäurer, Klaus Pfeifer

**Affiliations:** 1 Division Exercise and Health, Institute of Sport Science and Sport, Friedrich-Alexander-Universität Erlangen-Nürnberg, 91058 Erlangen, Germany; alexander.tallner@fau.de (A.T.); rene.streber@fau.de (R.S.); christian.hentschke@fau.de (C.H.); marc.morgott@fau.de (M.M.); Wolfgang.Geidl@fau.de (W.G.); klaus.pfeifer@fau.de (K.P.); 2 Department of Neurology, Stiftung Juliusspital, 97070 Würzburg, Germany

**Keywords:** multiple sclerosis, internet, telerehabilitation, resistance training, aerobic training, exercise, physical activity, health-related quality of life, fatigue, lung function

## Abstract

Physical exercise is effective in improving functional outcomes in persons with multiple sclerosis (pwMS). We evaluated the feasibility and effectiveness of internet-based exercise training (e-training) for pwMS on health-related quality of life (HrQoL). Secondary outcomes were muscle strength, aerobic capacity, lung function, physical activity, and fatigue. This is a randomised, controlled trial with a wait-list control group. Data were collected at baseline, after three and six months, and analysed using a hybrid linear model. One-hundred twenty-six pwMS participated in the home-based aerobic (1×/week) and strength training (2×/week) intervention that was supervised and documented via an internet-platform. The intervention group received e-training for six months, and the control group received e-training after a three months waiting period. Significant differences between the groups were only observed for muscle strength (knee flexion (effect size *ES* = 0.3, *p* = 0.003), knee extension (*ES* = 0.24, *p* = 0.015)), peak expiratory flow (*ES* = 0.2, *p* = 0.039), and sports activity (*ES* = 0.33, *p* = 0.001) after three months. E-training had no effect on HrQoL but did on muscle strength, lung function, and physical activity. It is a promising and feasible approach to facilitate large-scale, yet individual, training support.

## 1. Introduction

In the past, persons with multiple sclerosis (pwMS) were advised against physical activity [1]; however, the beneficial effects of physical activities and exercise [2] are now well documented. Nevertheless, major limitations of current exercise studies are short intervention periods (mainly 4–12 weeks) and small sample sizes (mainly between 10 and 50) [2,3]. One reason might be the location-dependent nature of interventions at a clinic or training centre [4]. Modern communication technologies via the internet have the potential to reach a large population over a wide area, allow economic delivery of an exercise intervention, and facilitate individually-tailored training support [5]. The objective of this study is to investigate the practicability and effectiveness of an internet-based exercise intervention including progressive strength and endurance training (e-training) for pwMS. The primary outcome was health-related quality of life (HrQoL), and secondary outcomes were muscle strength, aerobic capacity and lung function, physical activity, and fatigue.

## 2. Results

**Figure 1:**
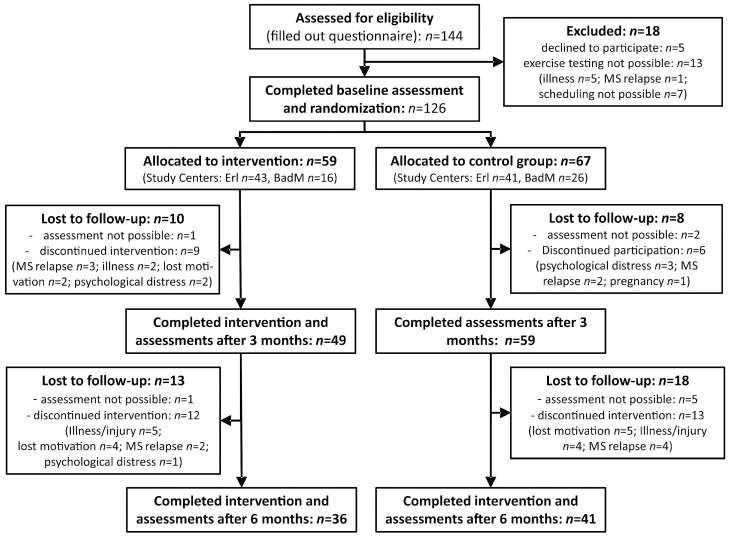
Flowchart of the study (study centres: Erl—Erlangen, BadM—Bad Mergentheim).

One-hundred twenty-six participants were randomised consecutively (Figure 1). After three months, the dropout rate amounted to 14% (*n* = 18, 10 in the intervention group, eight in the control group), and after six months to a total of 39% (*n* = 49).

Table 1 characterizes the sample at baseline. For the measured outcomes, significant baseline differences between intervention and control groups were only found for peak expiratory flow (*p* = 0.01). No differences were found for HrQoL (*p* = 0.462), fatigue (*p* = 0.407), aerobic fitness (*p* = 0.864), forced vital capacity (*p* = 0.929), knee extension (*p* = 0.594), knee flexion (*p* = 0.164), trunk extension (*p* = 0.988), trunk flexion (*p* = 0.825), and the sport score (*p* = 0.568).

### 2.1. Compliance

**Figure 2:**
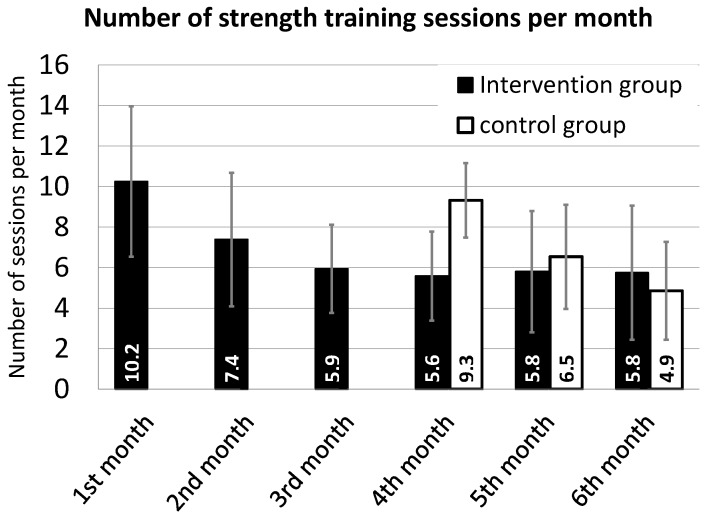
Compliance over the study period.

Over the course of the study, a total of 3639 strength training sessions with feedback on 21,566 individual training exercises were documented via the participant interface. A training plan contained 5.9 strength-building exercises on average. Figure 2 shows the number of strength training sessions per month of the participants who were included in the evaluation, which amounted to an average of 6.82 (*SD* = 2.72). A total of 8548 physical activities were recorded in the online activity journal. Apart from the strength training sessions, the most frequent entries were: cycling (*n* = 984), Nordic walking (*n* = 780), walking the dog (*n* = 605), running/jogging (*n* = 459), gym (*n* = 381), swimming (*n* = 333), walking (*n* = 209), and cross-trainer (*n* = 110). Regarding the frequency of use, 73% of participants in the intervention group documented at least 80% of the prescribed strength training sessions (two per week) during the first three months. A reduction in frequency of training gradually started after four weeks. In months 4–6, only 36% of participants documented at least 80% of the prescribed sessions.

### 2.2. Health-Related Quality of Life and Fatigue

Regarding HrQoL, no significant time effects within the groups or interaction effects were detectable in either the first or second phase of the study (Table 2 and Table 3). This holds true for the total score, as shown, but also for all the individual subscales of the questionnaire (data not shown).

There were also no significant effects for fatigue after three months, but in the second phase of the study the intervention group recorded a significant increase in fatigue.

### 2.3. Muscle Strength

The intervention group recorded significant increases in muscle strength after three months with a medium effect size for knee extension and knee flexion, which, in both cases were also significant in comparison with the control group. Significant increases in trunk flexion were recorded after three months for both the intervention group and the control group, which did not differ significantly between the groups. There was no change in trunk extension for either group in the first phase of the study, and in the second phase only the intervention group showed a significant increase in strength.

### 2.4. Aerobic Capacity and Lung Function

No significant changes were measurable for aerobic capacity, or static lung volume in either group. There was a significant increase in peak expiratory flow in the intervention group after thrree months of training, which was also significant in comparison with the control group.

### 2.5. Physical Activity

The intervention group recorded a highly significant increase in sports activities (Baecke sports score) after three months, while this increase became apparent in the control group in the second phase of the study, after training had begun.

## 3. Discussion

The e-training intervention as applied in this study could not influence the primary outcome HrQoL or fatigue, but secondary outcomes, such as muscle strength of the lower extremities, lung function and, especially, physical activity, improved significantly.

### 3.1. Health-Related Quality of Life

Effects of physical exercise training on HrQoL for pwMS have been demonstrated in two meta-analyses [6,7], but a more recent review [2] comes to the conclusion that an improvement in HrQoL through exercise is not assured. HrQoL for pwMS is a multidimensional construct that, according to existing reviews [7] can be positively influenced by endurance training rather than strength training, with the latter being mainly targeted by the e-training. Furthermore, the effects of exercise interventions on HrQoL could be mediated by social contact and support within the training group. This is reinforced by a study by Schulz et al. [8] in pwMS and a meta-analysis in the area of disease management [9]. Social support by peers was not present in the case of individual training at home in this study, which could explain the lack of effectiveness. It should, therefore, be investigated in future studies whether, and how, the various communication options offered by the internet (social media, online communities) can be used in a targeted way in order to create an equally highly perceived social support as it was seen in group training, and so possibly generate an effect on HrQoL.

### 3.2. Fatigue

Two review articles concluded that physical exercise training can improve fatigue [10,11]. However, effectiveness appears to be strongly dependent on whether the test group under investigation suffers from clinically-relevant fatigue [10]. This was not the case in the present study, as baseline values were markedly below the fatigue threshold. This might be the main reason for the lack of intervention effects on fatigue. The increase in fatigue in the intervention group in the second phase of the training is difficult to interpret. The progression of the exercise training load was not changed during this phase. Whether setbacks in the fatigue level would have been avoided as a result of a more varied training periodization with phases of changing (including decreasing) intensity is an interesting question for future investigation.

### 3.3. Muscle Strength

Isometric maximum strength of the knee extensors was increased by 9% and that of the knee flexors by 13% after three months of training. The effect sizes for leg strength gains accomplished in this study correspond well to the mean effect size of strength training in pwMS that was determined in a recent meta-analysis (mean *ES* = 0.27) [12]. This can be taken as evidence of the practicability and effectiveness of internet-supported strength training, particularly as higher training intensities and more precise training control are possible for supervised training with weights than for training at home. However, the control group did not reproduce the results of the intervention group—the reason for this could be lower training compliance (Figure 2), or a bias as a result of a higher incidence of dropout in the second phase of training compared to the first (*n* = 31 as against *n* = 18).

### 3.4. Aerobic Capacity and Lung Function

No effects on aerobic capacity could be achieved. A training-induced increase in Peak oxygen consumption in O_2_ mL/(kg·min) (VO_2peak_) of up to 10% of the measurement value is possible and realistic for pwMS [13]. Reasons for the lack of effect of the e-training on endurance could be low training frequency (one session per week) and the fact that the endurance training was not continuously progressed and difficult to standardize due to varying pre-existing levels of endurance training of the sample.

Nevertheless, the peak expiratory flow could be significantly increased as a result of the training. The baseline values of peak expiratory flow were approximately 84% of the norm, which may indicate a weakness of the respiratory musculature that occurs in pwMS at an early stage of the disease [14]. The reason for the effect on peak expiratory flow could, therefore, be a strengthening of the respiratory and auxiliary respiratory musculature [14,15].

### 3.5. Physical Activity

Physical activity improved significantly in both groups, corresponding with the respective start of the internet-supported training. The recorded mean change of about one point of the sport score corresponds to approximately 60 to 90 min of additional regular, moderately-intensive sports activity per week. This approximately meets the amount of prescribed exercise sessions of about 2–3 per week.

### 3.6. Compliance

Compliance and frequency of use are key aspects in the evaluation of internet-based interventions and deserve thorough analysis, as it is through this that the prescribed dosage of the intervention is determined. The frequency of use was higher than for most internet-based interventions [16]. This could be due to the fact that the software, as well as offering automated, interactive software features, also made contact with a therapist possible [17]. However, a reduction in documented training sessions that already gradually started after four weeks was observed. A pronounced decline in compliance has also been observed in other internet-based interventions with pwMS [18,19]. One reason could have been a decrease in motivation to log into the user interface and document completed training sessions. While frequency of use decreased over the course of time, the dropout rate increased (14% after three months and 39% after six months). This could have led to a bias, but the dropout rate is, nevertheless, similar to, or lower than, that for existing home training interventions for pwMS, which amounted to between 19% and 51% [20,21,22,23].

The presented study did not exploit the full potential of internet-based interventions and leaves room for improvement. An increase in the frequency of use and compliance, and a reduction in the dropout rate might be achieved by an increased level of support [17] or a stronger emphasis on the social aspects of training [24]. In addition to the technical and communicative possibilities of the internet [25,26], the application of theories, and models of behavioural change [27] considering needs, preferences, motives, and perceived barriers [28] of pwMS appears particularly promising to increase adherence and promote physical activity. Then, internet-based interventions may be a highly appropriate method of offering holistic, barrier-free, and customisable programs for physical activity promotion and long-term commitment to physical activity over a wide area.

### 3.7. Limitations

Although a number of measures were taken to prevent bias (standardization of assessment procedures, assessor training across study centers, blinding of assessors, and allocation concealment at baseline), we were not able to blind participants. Double-blinding is hardly possible in rehabilitation or training studies.

Another major disadvantage of the applied e-training platform is the lack of direct supervision. No cameras, motion detection systems, or other objective methods of observing the training were used. Non-functional execution of the exercises and faulty or incorrect training documentation are, therefore, not precluded.

Due to recruitment strategies, the sample was probably more active and predisposed to sports than average [29] and only pwMS with a maximum Expanded Disability Status Scale (EDSS) of 4.0 were included. Thus, the results cannot be generalised to inactive or more seriously affected persons. Those might be more suitable target groups for exercise interventions to improve quality of life.

## 4. Materials and Methods

### 4.1. Study Design

Approval for the study was given by the ethics committee of the medical faculty of the Friedrich-Alexander-University Erlangen-Nürnberg (Erlangen, Germany). The study was performed in two study centres and used a two-arm, randomised, controlled design with a wait-list control group and assessments at baseline, and after three and six months. The main effects of the e-training were assessed in the primary analysis after three months, by comparison of intervention to control conditions. To detect potential long-term effects, the intervention group received the e-training for another three months. The wait-list control group received no intervention for the first three months, and then three months of e-training. Both study centres applied identical and standardized procedures for assessment and intervention.

### 4.2. Subjects

Recruitment was carried out via contact lists from previous studies, press announcements in local newspapers, and websites of multiple sclerosis societies.

Criteria for inclusion were diagnosed multiple sclerosis, an Expanded Disability Status Scale (EDSS) score of less than or equal to 4.0, not less than four weeks of clinical stability prior to inclusion in the study, and access to the internet. Criteria for exclusion were primary progressive multiple sclerosis and clinically-relevant cardiological, internal, or orthopaedic contraindications to exercise, which were assessed by the patients’ attending physicians.

The randomisation was stratified according to the EDSS (cut-off value 2.5) and maximum oxygen uptake (VO_2peak_, cut-off value 30) as a measure of fitness. Allocation was carried out after the baseline data collection by entering the stratification criteria into an urn randomization program (uchc_urn.exe, available online: http://www.commed.uchc.edu/programs/health_services/match/urn/index.html). Thereby, allocation was concealed at the time of assessment.

### 4.3. Assessments

HrQoL was recorded with the Hamburg Quality of Life Questionnaire for Multiple Sclerosis (HAQUAMS) [30]. The total score is calculated from a mean of the subscales (fatigue/thinking, mobility, communication, mood), providing a score between 1 and 5. Fatigue was assessed by the Würzburg Fatigue Scale for Multiple Sclerosis (WEIMuS) [31]. The total score ranges from zero to a maximum of 68 points (maximum fatigue), with a cut-off value for the presence of fatigue of above 32.

Maximum isometric muscle strength was tested with the M3 Diagnos machine (Schnell, Peutenhausen, Germany). After a five-minute warm-up on a bicycle ergometer at 40 watts and familiarization (two submaximal trials) with the procedure, subjects were instructed to increase muscle contraction strength over a period of 1–2 s and then sustain maximum contraction for 5 s. Two repetitions with a rest interval of one minute were carried out. Peak torque values were calculated by the M3 Diagnosis software (Peutenhausen, Germany) and determined as the highest attained torque during one attempt. The best out of two attempts was recorded.

Forced vital capacity and peak expiratory flow were tested with the Master Screen CPX System (Viasys Healthcare, Cardinal Health, Höchberg, Germany) to evaluate lung function. After familiarization, the value of the best of two test attempts was taken. As a marker of endurance capacity, peak oxygen uptake (VO_2peak_) was determined by the Master Screen CPX System during a spiroergometry on a bicycle ergometer (Sanabike 250F, MESA, Benediktbeuern, Germany) at 70–80 revolutions per minute.

Habitual physical activity was assessed with the German version [32] of the Baecke Questionnaire including three activity indices (work, sport, leisure time, and physical activity). Psychometric testing of the German version showed good construct validity (correlation of the sport index with step counts and aerobic capacity) in pwMS [33]. Nevertheless, there were limitations concerning content validity in all indices; therefore, the sport score of the sport index was used. That score is the product of the intensity, duration, and frequency of a participant’s reported sports activities.

### 4.4. Intervention

After the initial assessment on entry, those assigned to the control group were instructed to maintain their previous physical activity behaviour. After waiting three months, they received the same e-training intervention as the intervention group had received from the start.

According to the recommendations available at the study design phase [3] and due to its good tolerability, strength training was declared core content of the intervention and prescribed twice weekly for a period of 12 weeks, with 2–3 sets per exercise. In addition, to facilitate effects on aerobic fitness and HrQoL, endurance training with one session per week was prescribed. The exercise training was home-based and supervised via the internet.

The e-training intervention began with a two-day, on-site training seminar on the content and procedures of the e-training. Afterwards, prescription and supervision of exercises and incorporation of participants’ feedback was organized via a browser-based software solution (motionNet e-Training, motionNET systems Ltd., Nürnberg, Germany) with one-to-one support for each participant. The trainers were exercise therapists and/or physical therapists with experience in the prevention and rehabilitation setting with different indications including multiple sclerosis. These therapists were additionally trained with regard to all study and prescription processes. Exercises were delivered with detailed descriptions and pictures in PDF documents. Training support and communication took place asynchronously (not in real-time) via a messaging service in the software and, when required, by email and telephone. No special equipment was necessary except an elastic exercise band or a large gymnastic ball. The number of sets and repetitions to be completed for each exercise were prescribed individually for each participant and training session, and were dependent on fitness levels. Training intensity was regulated by the participant’s subjective, perceived exertion, which was rated between 6 and 20 on the Borg Scale [34]. This scale is widespread in health-related exercise interventions and its validity for pwMS has been proven for both strength and endurance training [35]. Therapists aimed at eliciting a Borg Feedback of between 11 (fairly light) and 16 (hard). If successful, the next progression step was prescribed for the respective exercise. Between sets and exercises, a rest period of approximately 1–2 min was recommended. To ensure training overload and progression, we used a standardized progression scheme ranging from at least two times, six repetitions up to a maximum of three times, 20 repetitions. The progression scheme had an increment of two repetitions with altering numbers of sets (between two and three).

In addition to the strength training, endurance training was to be carried out once a week. Based on the spiroergometric evaluation, recommendations regarding the intensity of jogging, walking, cycling, and swimming were made (V-Slope method, intensity slightly above the first ventilatory threshold). The form of activity for the endurance training was freely selected, duration (between 10–60 min) was adjusted to individual fitness levels. In contrast to the strength training, the endurance training was, however, not systematically progressed after the initial recommendation of training parameters. A more detailed intervention description is available on the clinicaltrials.gov entry [36] for the study (NCT02771652, study documents).

Compliance with the e-training was recorded electronically via the documentation function of the training software.

### 4.5. Statistical Procedures

A sample size calculation determining a significance level of 0.05, statistical power of 0.8, medium effect size (*f*(*V*) = 0.25), and two groups and two measurements (within-between interaction) yielded *n* = 128. Taking into account a drop-out rate of approximately 35%, the required sample size was *n* = 172.

Changes in the intervention group and the control group were calculated with a hybrid linear model that was based on a 3 × 2 factorial design and included all three measurement times (T0, T1, T2) and both intervention groups as fixed effects, and the intercept and slope as random effects. Primary analysis was at T1, after three months’ intervention, against the control group. Changes in the measured values were treated and analysed individually for each time period. In this connection, changes in the groups with respect to the value of the previous measurement were determined and tested for statistical significance both within the group (time effect) and in a comparison of the groups (interaction group × time). Through this procedure, differences arising at the previous measurement (and, thereby, baseline differences) were statistically controlled. Grouping by time effects, though, were only calculated for the primary analysis at T1, since this comparison is not meaningful for T2. Furthermore, all outcomes were adjusted according to the baseline value of the EDSS. All available observations were included in the analysis as randomised (full information likelihood), there was no imputation of missing data. The results are therefore not biased by a missing at random (MAR) assumption. Effect sizes were calculated for time effects and for group × time interactions (Cohen’s d) and interpreted as large (>0.8), medium (0.5–0.8) or small (0.2–0.5) effects.

## 5. Conclusions

Internet-based exercise interventions can lead to improvements in physical activity levels and physical function outcomes in persons with multiple sclerosis. This e-training intervention had no effect on health-related quality of life but is a promising and feasible approach to facilitate individually-tailored training support, considering the large sample size and significant effects on muscle strength, lung function, and sports activities. Reduction of compliance over time is a shortcoming, especially of internet-based studies, and needs to be explored and systematically counteracted in further studies. To improve adherence and health-related quality of life, social support could to be an important factor to be considered in future exercise interventions.

## Figures and Tables

**Table 1:** Baseline characteristics of the study participants by group (mean (SD) or *n* (%)).

Title	Intervention Group (*n* = 59)	Control Group (*n* = 67)	Total Sample (*n* = 126)	Difference Between Groups ^a^
Number				
Female	44 (75%)	50 (75%)	94 (75%)	NA
Male	15 (25%)	17 (25%)	32 (25%)	NA
Type of Multiple Sclerosis				
relapsing-remitting	52 (88%)	57 (85%)	109 (87%)	NA
secondary-progressive	7 (12%)	10 (15%)	17 (13%)	NA
Age (Range 16–76)	40.9 (10.4)	40.7 (9.5)	40.8 (9.9)	*p* = 0.895
Duration of disease	9.8 (9.2)	9.2 (7.2)	9.5 (8.2)	*p* = 0.651
EDSS	2.8 (0.8)	2.7 (0.8)	2.7 (0.8)	*p* = 0.529

^a^—Based on *t*-test; EDSS—Expanded Disability Status Scale; NA—Not applicable.

**Table 2:** Primary and secondary outcomes by group (mean (*SD*)) at baseline, three-month, and six-month assessment.

Outcome	Intervention Group			Control Group		
	Baseline	Three-Month	Six-Month	Baseline	Three-Month	Six-Month
HRQoL	1.84 (0.42)	1.85 (0.49)	1.87 (0.49)	1.79 (0.4)	1.83 (0.42)	1.78 (0.43)
Fatigue	21.3 (12.9)	19.05 (14.4)	23.87 (15.0)	23.5 (16.3)	21.93 (15.3)	20.56 (14.9)
Aerobic fitness (VO_2peak_)	25.3 (6.1)	25.66 (5.39)	25.32 (6.63)	25.2 (6.5)	24.91 (6.29)	25.51 (6.68)
Forced vital capacity (L)	4.0 (0.9)	4.18 (0.86)	4.17 (0.77)	4.18 (0.96)	4.17 (0.99)	4.22 (1.00)
Peak expiratory flow (L/sec)	6.0 (1.53)	6.41 (1.43)	6.54 (1.56)	6.78 (1.69)	6.72 (1.59)	6.94 (1.58)
Knee extension (Nm)	287 (100)	302.81 (93)	311.1 (100)	298 (118)	292.98 (113)	292.07 (111)
Knee flexion (Nm)	156 (50)	177.71 (59)	181.45 (52)	171 (65)	175.3 (63)	179.05 (62)
Trunk extension (Nm)	169 (62)	169.88 (59)	181.66 (58)	168 (67)	169.26 (72)	173.24 (69)
Trunk flexion (Nm)	86 (32)	91.41 (32)	95.17 (31)	85 (36)	88.32 (38)	91.07 (39)
Sport score	2.3 (2.47)	3.24 (2.73)	3.36 (2.33)	2.1 (2.43)	1.9 (2.39)	2.86 (2.89)

HRQoL—Health-related quality of life; L—litres; Nm—Newton metre; VO_2peak_—Peak oxygen consumption in O_2_ mL/(kg·min).

**Table 3:** Time effects and between group effects at three and six months after baseline assessment.

Outcome	Intervention Group (Time Effect)		Control Group (Time Effect)		Between Group Difference (Group × Time)	
	Mean Change [95% CI]	*ES*, *p*-Value	Mean Change [95% CI]	*ES*, *p*-Value	Difference of Mean Changes ^a^ [95% CI]	*ES*, *p*-Value
HRQoL						
Three-month	0.02 [−0.06, 0.11]	0.08, *p* = 0.58	0.03 [−0.04, 0.11]	0.11, *p* =0.40	−0.01 [−0.12, 0.10]	−0.01, *p* = 0.88
Six-month	0.04 [−0.06, 0.13]	0.13, *p* = 0.45	−0.05 [−0.14, 0.03]	−0.18, *p* = 0.25		
Fatigue						
Three-month	−2.06 [−5.74, 1.62]	−0.16, *p* = 0.28	−1.81 [−5.11, 1.49]	−0.14, *p* = 0.29	−0.25 [−5.19, 4.69]	−0.01, *p* = 0.92
Six-month	5.3 [1.15, 9.46]	0.42, *p* = 0.02	−1.3 [−5.03, 2.43]	−0.1, *p* = 0.50		
Aerobic fitness (VO_2peak_)						
Three-month	0.13 [−0.83, 1.08]	0.04, *p* = 0.80	−0.33 [−1.19, 0.54]	−0.1, *p* = 0.46	0.45 [−0.84, 1.74]	0.07, *p* = 0.49
Six-month	−0.34 [−1.46, 0.78]	−0.11, *p* = 0.55	0.71 [−0.28, 1.71]	0.23, *p* = 0.17		
Forced vital capacity (L)						
Three-month	−0.03 [−0.12, 0.05]	−0.11, *p* = 0.46	−0.02 [−0.1, 0.06]	−0.06, *p* = 0.657	−0.01 [−0.13, 0.1]	−0.02, *p* = 0.81
Six-month	−0.01 [−0.11, 0.09]	−0.03, *p* = 0.84	0.05 [−0.04, 0.14]	0.19, *p* = 0.25		
Peak expiratory flow (L/sec)						
Three-month	0.36 [0.07, 0.65]	0.35, *p* = 0.02	−0.06 [−0.33, 0.21]	−0.06, *p* = 0.65	0.42 [0.03, 0.82]	0.2, *p* = 0.04
Six-month	0.12 [−0.22, 0.46]	0.12, *p* = 0.49	0.23 [-0.08, 0.53]	0.23, *p* = 0.16		
Knee extension (Nm)						
Three-month	20.52 [7.56, 33.48]	0.46, *p* = 0.003	−1.39 [−12.91, 10.13]	−0.03, *p*=0.81	21.91 [4.57, 39.24]	0.24, *p* = 0.02
Six-month	5.11 [−9.96, 20.18]	0.11, *p* = 0.51	0.04 [−13.29, 13.38]	0.0, *p* = 0.99		
Knee flexion (Nm)						
Three-month	19.61 [12.03, 27.19]	0.76, *p* = 0.00	3.62 [−3.11, 10.35]	0.14, *p* = 0.30	16 [5.86, 26.13]	0.3, *p* = 0.003
Six-month	2.34 [−6.39, 11.07]	0.09, *p* = 0.60	4.17 [−3.55, 11.89]	0.16, *p* = 0.30		
Trunk extension (Nm)						
Three-month	−1.11 [−9.53, 7.31]	−0.04, *p* = 0.80	0.02 [−7.54, 7.57]	0, *p* = 0.99	−1.13 [−12.44, 10.18]	−0.02, *p* = 0.85
Six-month	10.21 [0.56, 19.86]	0.36, *p* = 0.05	4.46 [−4.1, 13.02]	0.16, *p* = 0.31		
Trunk flexion (Nm)						
Three-month	6.29 [2.65, 9.93]	0.5, *p* = 0.001	3.95 [0.69, 7.21]	0.31, *p* = 0.02	2.34 [−2.55, 7.22]	0.09, *p* = 0.35
Six-month	3.2 [−1.01, 7.41]	0.26, *p* = 0.15	2.93 [-0.8, 6.66]	0.24, *p* = 0.13		
Sport score						
Three-month	0.91 [0.44, 1.37]	0.55, *p* = 0.00	−0.19 [−0.61, 0.24]	−0.11, *p* = 0.393	1.09 [0.46, 1.73]	0.33, *p* = 0.001
Six-month	0.12 [−0.41, 0.66]	0.08, *p* = 0.65	0.96 [0.47, 1.45]	0.59, *p* = 0.00		

^a^—higher values display positive changes in favor of the intervention group, except for HrQoL and Fatigue scales; ES—standardized effect size; HRQoL—Health-related quality of life; L—liters; Nm—newton metre; VO_2peak_—peak oxygen consumption in O_2_ mL/(kg·min).
